# Insights into the structure and evolution of the human SAGA complex by affinity-ligand purification

**DOI:** 10.1126/sciadv.aec8104

**Published:** 2026-03-18

**Authors:** Mylène Damilot, Thomas Schoeps, Laszlo Tora, Patrick Schultz, Luc Lebeau, Gabor Papai, Adam Ben-Shem

**Affiliations:** ^1^ Department of Integrated Structural Biology, Institut de Génétique et de Biologie Moléculaire et Cellulaire (IGBMC), Illkirch, France.; ^2^ Université de Strasbourg, Illkirch, France.; ^3^ CNRS, UMR 7104, Illkirch, France.; ^4^ Inserm, UMR S 1258, Illkirch, France.; ^5^ Equipe labellisée Ligue Contre le Cancer, Institut de Génétique et de Biologie Moléculaire et Cellulaire (IGBMC), Illkirch, France.; ^6^ Department of Functional Genomics and Cancer, Institut de Génétique et de Biologie Moléculaire et Cellulaire (IGBMC), Illkirch, France.; ^7^ Laboratoire de Chémo-Biologie Synthétique and Thérapeutique, UMR 7199, CNRS-Université de Strasbourg, Illkirch, France.

## Abstract

Human SAGA is a 20-subunit transcriptional coactivator. Compared with yeast, metazoan SAGA uniquely incorporates a 150-kDa splicing-factor module (SPL), also present in U2 small nuclear ribonucleoprotein (U2snRNP). Metazoan gene duplication further specialized shared TFIID/SAGA subunits into SAGA-specific paralogs (TAF5L and TAF6L), but the functional consequences of this divergence are unknown. We report the structure of endogenous human SAGA purified via an affinity ligand from cells that were not disturbed by any genomic engineering tools. Our work reveals the high-resolution structure of SPL and the TAF6L HEAT repeat domain that provides the SPL with a docking surface. We elucidate how SPL and the HEAT repeats are incorporated into SAGA. We identify major structural differences between TAF6L/TAF5L and their canonical paralogs that enable SPL accommodation. SPL engages SAGA through a substantially smaller interface than in U2snRNP, despite sharing a deeply inserted helical motif. The seemingly weaker interaction of SPL with SAGA raises the possibility that SAGA relays this module to the splicing machinery.

## Teaser

Structural insights explain how human SAGA evolved to host a splicing module absent in yeast.

## INTRODUCTION

Transcription regulation controls many cell decisions including growth, differentiation, and response to external cues. DNA sequence–specific transcription factors recruit coactivators/repressors, many of them are large multiprotein complexes, to stimulate or inhibit transcription by altering chromatin structure. SAGA is such a large complex that modulates chromatin structure via two enzymatic activities: histone acetylation and deubiquitination (*1*).

Human SAGA regulates both transcription initiation and elongation and is essential for normal embryonic development (*2*). It also has many nonhistone targets and thus affects gene expression at multiple levels from transcription to protein stability (*3*, *4*). The ~1.6-MDa complex comprises 20 subunits organized functionally and structurally in five modules: a histone acetyltransferase (HAT) module, a histone deubiquitinase (DUB) module, the TRRAP subunit that serves as a docking surface for transcription activators that recruit SAGA to enhancers, a metazoan-specific 150-kDa splicing-factor module (SPL) with an enigmatic role, and a central module that scaffolds the complex by physically connecting to all other modules. Cryo–electron microscopy (cryo-EM) maps of human and yeast SAGA describe well the core and TRRAP parts but do not reveal at high resolution the HAT, DUB, or splicing modules (*5*–*7*).

GCN5/PCAF is the catalytic subunit of the SAGA HAT module. This subunit is also incorporated into the essential 10-subunit coactivator ATAC, where the HAT module is identical to that of SAGA with the exception of one subunit being replaced by a homolog. SAGA and ATAC are regulating distinct sets of genes (*8*).

In yeast, the SAGA core module shares five subunits of the TATA box–binding protein (TBP) depositing complex TFIID: Taf5, Taf6, Taf9, Taf10, and Taf12. In metazoans, gene duplication created TAF6 and TAF5 paralogs that are specific to SAGA, namely, TAF5L and TAF6L. It is currently poorly understood why gene duplication was necessary. What specialized roles do the differences between TAF5L/TAF6L and their yeast or TFIID counterparts play? Are these roles related to the most prominent metazoan-specific feature of SAGA, the splicing module? Answering these questions is hindered by the low-resolution description of the splicing module as well as its docking site on SAGA.

In line with their pivotal role in transcription regulation, many coactivators are implicated in human disease, notably cancers. For example, SAGA can be recruited to chromatin by the c-MYC oncoprotein whose deregulation leads to unfavorable variations in the expression of target genes in the vast majority of cancers. The HAT activity of SAGA is necessary for the maintenance of MYC oncogenic transcription program in many tumors (*9*). Given the involvement of SAGA in human pathology, there is an urgent need to characterize the composition, interactome, activity, and structure of endogenous SAGA in cell lines derived from tumors or other human diseases. However, this remains a largely untapped source of information, due to the difficulty in purifying these complexes. Although affinity tags fused to endogenous subunits of SAGA via CRISPR-Cas9 engineering could alleviate this issue, many cell lines are not amenable to this technology. Moreover, the efforts that such tagging entails impede large comparative studies where SAGA from several different cell lines is required. Furthermore, optimized purification schemes are required due to the very low abundance of these complexes in cells. For example, a recent study reported only a few picomoles of isolated SAGA from 30 liters of HeLa cell culture (*5*).

Affinity ligands, small molecules that bind SAGA and can be coupled to a solid support (e.g., agarose or magnetic beads), offer an attractive alternative to affinity tags because they can be applied to many different cell lines. Several small molecules/inhibitors are available that target with high affinity and selectivity the active site of SAGA or its “reader” domains that stabilize interactions with chromatin through recognizing specific histone modifications. However, so far, such molecules were not exploited for developing a purification scheme that yields pure and active SAGA, or any other low-abundance coactivator, let alone enables their structure determination.

In this study, we obtained pure and highly active endogenous human SAGA from two different unmodified cell lines, K562 and HeLa, using an affinity ligand. This compound is composed of an inhibitor for the bromodomain of the enzymatic subunit GCN5/PCAF in the SAGA HAT module and is coupled to desthiobiotin. We introduced several innovations in the design of the affinity ligand as well as in the purification scheme that was adapted to low-abundance complexes. Using this method, we determined the structure of the purified SAGA by cryo-EM and revealed at high-resolution the SPL in SAGA and as well as the TAF6L HEAT repeats, which form the docking site for the splicing and HAT modules. We elucidated in detail how the splicing module and TAF6L HEAT repeats anchor into the coactivator complex. We find that the major deviations in TAF6L with respect to the canonical paralog are required for incorporating the splicing module. Comparing the docking of this module in SAGA and in the spliceosome suggests that SAGA serves to relay the splicing module to its assembly in the spliceosome. Our results could potentially guide future endeavors to characterize multiprotein complexes from many medically important sources using affinity ligands.

## RESULTS

### Design of the affinity ligand and the purification scheme

**Fig. 1.**
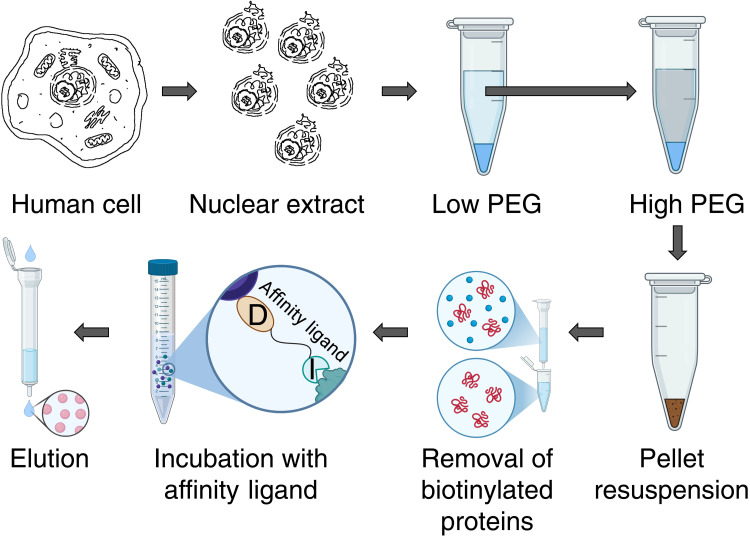
Purification scheme of SAGA and ATAC using affinity ligand. Schematic overview illustrating the stepwise purification strategy adapted to low-abundance complexes used to isolate SAGA and ATAC. Low and high PEG, precipitation with low and high concentration of PEG; I, inhibitor; D, desthiobiotin. A part of the figure was created in BioRender. G. Papai (2026); https://BioRender.com/4ybo9xk.

Low-abundance coactivators, such as SAGA, are present at a concentration of roughly 1 nM in nuclear extracts. On the other hand, the majority of inhibitors for catalytic enzymes or for histone modification “readers” harbored by coactivators have a dissociation constant (*K*_d_) higher than the very low–nanomolar range. Hence, the first obstacle that we needed to overcome, in order to make the use of most available inhibitors feasible, was to develop a method for concentrating nuclear extracts. Our experience in yeast and human cells showed that differential precipitation using a high–molecular weight polyethylene glycol (PEG) is a simple technique to achieve that goal (Fig. 1). Due to PEG tendency to precipitate first the larger molecules, this technique can also be considered as a real first purification step because it increases the proportion of large complexes in the sample with minimal losses (*10*). We worked with nuclear extract derived from 3 liters of K562 or 4.5 liters of HeLa S3 cells in suspension due to sample requirements for high-resolution structural studies. However, much smaller volumes can be used for other applications, since PEG precipitation procedure can be easily adapted to practically any volume of nuclear extract.

We then screened the literature for high-affinity and selectivity binders for SAGA. We eventually chose GSK4027 (*11*), which targets the bromodomain, a “reader” that recognizes acetylated lysines, in the catalytic HAT subunit GCN5/PCAF with a *K*_d_ = 1.4 nM (fig. S1A). This compound is highly selective to GCN5/PCAF, presenting much lower affinity to other bromodomains. One drawback of using GSK4027 as a ligand is that it cannot distinguish between the two human complexes that harbor GCN5/PCAF, namely, ATAC and SAGA. However, we posited that due to the predicted size difference between SAGA (1.68 MDa) and ATAC (0.9 MDa), in silico classification of the cryo-EM dataset would be able to separate the two complexes.

To mediate the binding of GSK4027 to a solid matrix, we chose to conjugate it to desthiobiotin, which can be attached to streptavidin beads (*K*_d_ = 10^−11^ M) and efficiently eluted under native conditions with biotin (*K*_d_ = 10^−16^ M). From x-ray crystallographic data on GSK4027 bound to the human GCN5/PCAF bromodomain, the 4-position on the pendant phenyl ring seems to point outside the binding pocket (*11*). This is confirmed by results obtained by Bassi *et al.* who developed a GSK4027-based PROTAC approach for modulating GCN5/PCAF immune cell function (*12*). This position was thus selected for derivatization with desthiobiotin while preserving binding to the bromodomain. Consequently, we designed conjugates 1a and 1b as ligands for immobilization of SAGA/ATAC on streptavidin beads and purification by affinity chromatography (fig. S1B). The GSK4027 and desthiobiotin moieties are connected through an oligoethylene spacer of variable length to modulate access of each ligand to its respective target, GSK4027 to the bromodomain and desthiobiotin to streptavidin beads, and alleviate possible steric hindrance.

### Synthesis of the affinity ligands 1a and 1b

Prior to our work, the synthesis of GSK4027 was achieved by Humphreys *et al.* in seven steps (*11*). The compound was obtained as a mixture of four enantiomers, and final separation of the racemate by preparative chiral high-performance liquid chromatography was necessary to get the desired single enantiomer in an overall yield of 1.2%. In this study, we developed a stereocontrolled synthetic route to prevent the formation of mixed isomers. This approach aligns with a methodology previously established by Van Steijvoort *et al.* (*13*) involving a Pd-catalyzed stereoselective C5(sp^3^)-H arylation of 1-Boc-3-(picolinoylamino)piperidine with an aryl iodide (*13*). The synthesis of the GSK4027 scaffold (compound 7) is a six-step process with an overall yield of 7.6% (fig. S1C). Two additional steps are required for the target compounds 1a and 1b. The full experimental details are provided in Materials and Methods.

### Purification of active SAGA/ATAC using affinity ligand

**Fig. 2.**
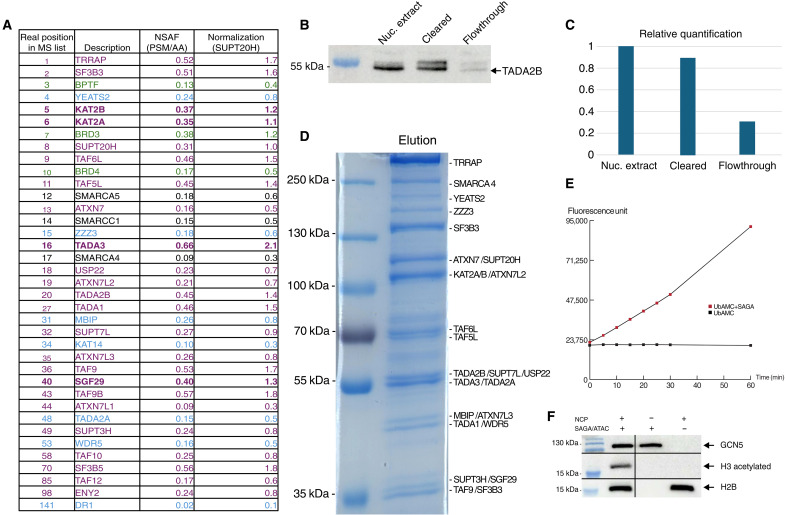
Purification of SAGA/ATAC using affinity ligand with long linker. (**A**) Proteomic analysis of the purified SAGA and ATAC complexes. For each identified protein the table shows the normalized spectral abundance factor (NSAF) value calculated from the PSMs divided by the number of amino acids (AA), and the normalization of the NSAF value to SUPT20H as rough estimation of stoichiometry. SAGA subunits are colored purple, ATAC subunits are colored blue, purple bold represents the common subunits between SAGA and ATAC, and copurified bromodomain containing proteins are in green. (**B**) Western blot analysis of different purification steps detecting the TADA2B subunit of SAGA. (**C**) Relative quantification of the detected bands in (B). (**D**) Colloidal Coomassie blue–stained SDS-PAGE of the purified SAGA and ATAC complexes; the identity of indicated bands was verified by MS. (**E**) Deubiquitination activity of the purified SAGA on the fluorogenic ubiquitin–amino methyl coumarin (Ub-AMC) over time. (**F**) Acetyltransferase activity of the purified SAGA and ATAC complex on nucleosome core particles (NCPs). Acetylation of histone was detected by a pan-acetyl-lysine antibody. Anti-GCN5 and histone H2B antibodies were used as loading control of SAGA/ATAC and NCPs, respectively.

Nuclear extracts from cell lines K562 or HeLa S3 are first treated with a very low (~1%) concentration of PEG 20,000, to deplete the extracts from large particles such as membrane parts and vesicles. A higher concentration (~5%) of PEG is then used to pellet large multiprotein complexes including SAGA and ATAC. The pellet is suspended in a small volume that is found to contain almost all SAGA/ATAC particles but only 30 to 40% of the total starting protein content (Fig. 2, A to C). Hence, the PEG precipitation step concentrates the sample by ~20 times with minimal loss and, at the same time, increases the proportion of the target complexes. We obtained similar results with other large complexes (*10*).

We analyzed the eluted sample derived from wild-type K562 cells by SDS–polyacrylamide gel electrophoresis (SDS-PAGE) and mass spectrometry (MS) (Fig. 2, A and D). The isolated complex is ~90% pure as estimated by a polyacrylamide gel that also shows all known subunits of SAGA and ATAC with the exception of the very small ones (Fig. 2D). Similarly, MS analysis identifies all predicted subunits of SAGA and ATAC (Fig. 2A). Moreover, roughly 60% of the total MS signal, estimated by summing all peptide spectrum matches (PSMs), corresponds to subunits of SAGA and ATAC complexes. This high value is close to that obtained by tandem-tag affinity purification of low-abundance complexes yielding high-resolution cryo-EM structures (*10*). We note that purity estimation by MS is always much lower than SDS-PAGE due to its high sensitivity. Among the 20 subunits with the highest signal in the MS data, only six are not attributed to SAGA/ATAC (Fig. 2A). Three of the six are bromodomain-containing proteins (BPTF, BRD3, and BRD4) and probably present some specific affinity for the GSK4027 ligand. However, compared to SAGA, these are small proteins that will be easily removed in the cryo-EM data analysis, and their stoichiometry in the MS table is lower than SAGA subunits.

SDS-PAGE and MS analysis of eluted sample derived from HeLa S3 cells showed similar results demonstrating the versatility of our purification method that can be applied to different unmodified cell lines (fig. S2). We elected to continue our investigation only with the K562 cells because the SAGA concentrations seemed higher in this source.

To understand the importance of the linker that connects desthiobiotin to GSK4027, we compared purification that uses ligand 1a (i.e., with a linker made of three ethylene glycol units) instead of 1b. We find that linker length has a profound and critical effect on the quality of the purification. Using the conjugate with the shorter linker captures only a small fraction of SAGA/ATAC on the beads and yields a highly contaminated sample (fig. S3).

From the MS data, some interesting insight could be gleaned regarding the stoichiometry of the different subunits of ATAC complex. It seems that at least two subunits, namely, YEATS2 and MBIP, are present at two copies in the ATAC complex. In addition, we find that K562 cells host roughly three to four times more SAGA particles than ATAC. At least in this human cell line, the major agent of H3 acetylation is likely to be SAGA.

To further demonstrate the quality of SAGA/ATAC purified using our affinity ligand, we performed activity assays. First, we tested the capacity of SAGA to cleave immediately after a ubiquitin using the fluorogenic substrate, ubiquitin–amino methyl coumarin (Ub-AMC) (*14*). As Fig. 2E demonstrates, SAGA is highly active in cleavage of Ub-AMC, releasing the AMC moiety from ubiquitin and thus giving rise to a strong fluorescence signal. Second, we incubated SAGA/ATAC with nucleosomes in the presence of acetyl–coenzyme A (acetyl-CoA) and followed the acetylation of histones using an antibody that recognizes acetylated lysines. It seems that SAGA/ATAC is highly active in acetylating histone H3 (Fig. 2F). Clearly, we cannot exclude the possibility that only one of the two complexes is active, but this seems unlikely as both were purified together, harbored a nearly identical HAT module, and appeared intact in MS and SDS-PAGE.

Using PEG clearance and concentration of nuclear extracts as a starting point to a purification that uses an affinity ligand conjugated via a long (six ethylene glycol units) linker to desthiobiotin, we are able to isolate highly pure and active native tag-less coactivators in relatively large amounts (~20 μg of SAGA obtained from K562 cells and ~15 μg from HeLa cells).

### Structure of SAGA—Overview

**Fig. 3.**
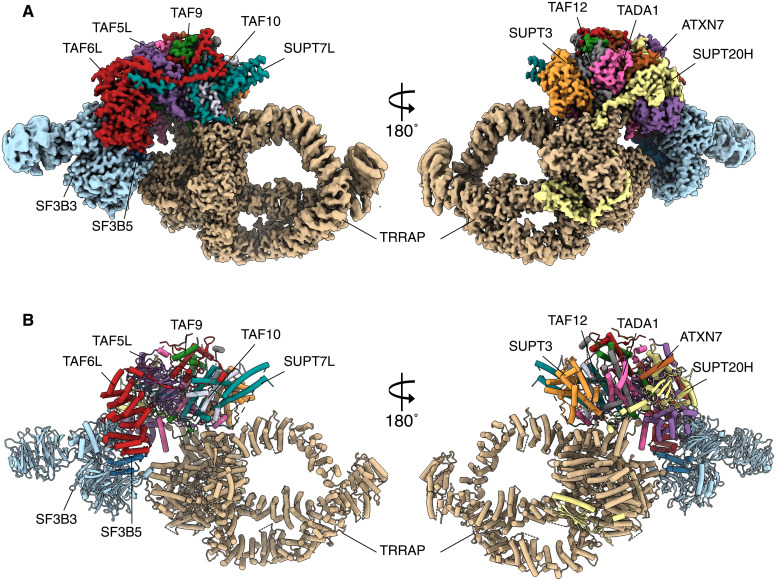
Structure of the human SAGA complex purified by long-linker affinity ligand. (**A**) Two views of the composite cryo-EM map of human SAGA. (**B**) Corresponding views of the atomic model of the SAGA complex.

We elucidated the structure of SAGA at 2.4- to 3-Å resolution (fig. S4). Our structure of human SAGA supports earlier descriptions of the complex. Human SAGA has a three-lobed architecture: One lobe consists mainly of the TRRAP activator-platform subunit, a second includes a Y-shaped splicing module, and the third, core lobe, is orchestrated by the TAF5 WD40 domain and contains a deformed octamer of histone folds (Fig. 3). As noted by others, the major deviation between human and yeast SAGA, apart from the additional splicing module, is the rotation of the TRRAP subunit around the core by nearly 80° (*5*). Furthermore, the interaction between the core and TRRAP is much more extensive in the human case where most core subunits contribute to the contact compared to only three in yeast (*6*).

Two additional lobes appear in the yeast three-dimensional (3D) maps of SAGA at very low resolutions and correspond to the HAT and DUB modules that seem to maintain some contact between themselves, as is suggested by cross-linking data as well (*15*). In the 3D maps of human SAGA, we find no trace of HAT and DUB, although MS strongly indicates that they are attached to SAGA, and moreover, the complex is purified via a ligand that recognizes the HAT module. This would suggest that the flexibility of these modules in the human case is enhanced compared to yeast. In yeast, the DUB module associates with the core through the N-terminal domain (NTD) of TAF5, and the HAT seems to have some ties to Spt7 (*16*). In the human counterpart, however, TAF5L NTD is translocated to a different position to vacate space for the splicing module (*5*). Similarly, human Spt7 (SUPT7L) is shorter by 600 residues than its yeast homolog and has much weaker, if any, contact with the HAT module. Hence, the loss of contacts with the core could underlie an enhanced autonomy or flexibility for the HAT and DUB modules.

### The splicing module in SAGA

Earlier studies of human SAGA (*5*, *17*) could visualize the structure of the 150-kDa splicing module only at low resolution and model it via rigid body docking of existing structure. This module, composed of the SF3B3 and SF3B5 factors, is absent in yeast SAGA. These earlier studies also failed to define in high resolution the TAF6L HEAT repeats, which are of crucial importance in SAGA because they are the docking site of the HAT and splicing modules and provide their connection to the core. Therefore, a high-resolution understanding of how the splicing module docks on SAGA and how the TAF6L HEAT repeats are incorporated into the core is lacking. The structure of endogenous human SAGA purified by affinity ligand can fill this gap in our understanding.

**Fig. 4.**
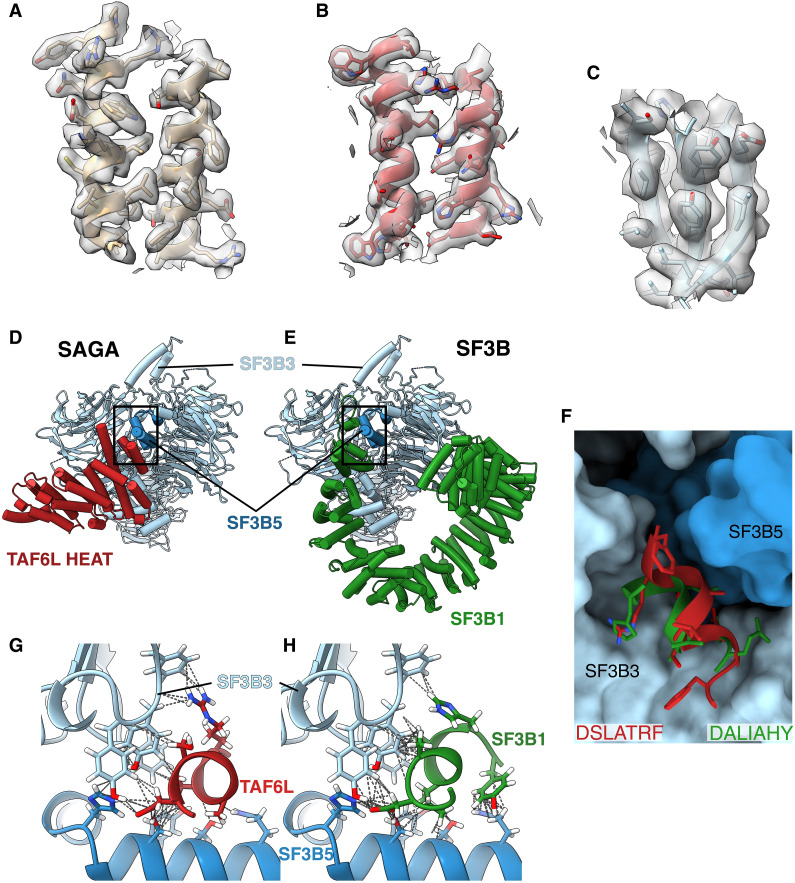
Splicing factor SF3B interaction with SAGA complex. (**A** to **C**) Representative cryo-EM densities showing the map quality in the TRRAP, TAF6L HEAT repeats, and SF3B regions, respectively. (**D**) Interaction between SF3B3 (light blue)–SF3B5 (dark blue) splicing module with TAF6L HEAT repeats (red) in human SAGA. (**E**) Interaction between SF3B3 (light blue)–SF3B5 (dark blue) splicing module with SF3B1 (green) in SF3B. (**F**) Close-up view of the homologous last helix in TAF6L HEAT repeats (red) and SF3B1 (green) interacting with a surface formed by SF3B3 (light blue surface) and SF3B5 (dark blue surface). (**G**) Interaction between the last helix in TAF6L HEAT repeats (red) and SPL (SF3B3, light blue; SF3B5, dark blue). The contacts are shown with dotted lines. (**H**) Interaction between the last helix in SF3B1 HEAT repeats (green) and SPL (SF3B3, light blue; SF3B5, dark blue). The contacts are shown with dotted lines.

We obtained maps with a resolution of roughly 3 Å on the splicing factors and TAF6L HEAT repeats, which allowed us to analyze their interaction at the level of side chains (Fig. 4, A to C). The SF3B3 and SF3B5 factors also associates with protein SF3B1 in the SF3B complex, an intrinsic component of the functional U2 small nuclear ribonucleoprotein (U2snRNP), part of the mRNA splicing machinery. We could therefore compare the TAF6L-SPL contacts in SAGA to the association of SPL with SF3B1 in the SF3B complex (Fig. 4, D and E). We find that the partners of the splicing module, namely, SF3B1 and TAF6L HEAT repeats, form a right-handed and left-handed super helix, respectively (Fig. 4, D and E). This inherent difference in their architecture results in little overlap between the positions of their HEAT repeat helices with respect to SPL. The networks of interactions in the two cases are different in general and have little in common. However, two similarities stand out. First, both TAF6L HEAT repeats and SF3B1 interact with the same face of SPL and are therefore mutually exclusive. Second, the last HEAT repeat helix of both SF3B1 and TAF6L are both located in the same place with respect to SPL and contribute considerably to the interaction with SF3B3 and SF3B5 (Fig. 4F). Furthermore, we find that the tip of this helix, namely, the last seven residues, have very similar sequences in both cases (DSLATRF in TAF6L and DALIAHY in SF3B1) and establish similar interactions with SPL (Fig. 4, G and H).

Notably, in SF3B1, this last helix is continued by a 30–amino acid linker that is inserted deep into a cleft between SF3B5 and SF3B3, interacting with both proteins and probably playing an important role in stabilizing the interaction in SF3B. Overall, we find that the buried surface involved in the interaction between TAF6L and SPL in SAGA is only half of that between SF3B1 and SPL in SF3B (1500 Å^2^ versus 3000 Å^2^). This suggests that SAGA binds SPL much weaker than the functional U2snRNP.

Another noteworthy observation regarding the SPL is the location of the seven-bladed β-propeller (BP) domain known as BPB (residues 472 to 770). We find that the position of this domain with respect to the rest of the module is shifted by 20 Å when compared to the crystal structure of the SF3B complex (*18*). This position is similar to the position of BPB within the spliceosome (*19*). This suggests that a similar conformation change accompanies loading of SPL into SAGA or spliceosome.

### Incorporating the TAF6 HEAT repeats—Importance of gene duplication creating SAGA specialized genes

TAF6L HEAT repeats are a structural hub that coordinates the HAT and splicing module and connects them to the core. In yeast, the TAF5 NTD and the SPT7 NTD play an important role in stabilizing the TAF6 HEAT repeats (*6*, *7*). However, in the human case, TAF5L NTD is displaced by SPL, and SUPT7L NTD is just a short linker that only weakly, if at all, contributes to binding the HEAT repeats. These changes were probably required for adding the splicing module and affording greater autonomy to the HAT, but what evolutionary adjustments made these changes possible while still maintaining TAF6L HEAT repeats well docked into the core?

**Fig. 5.**
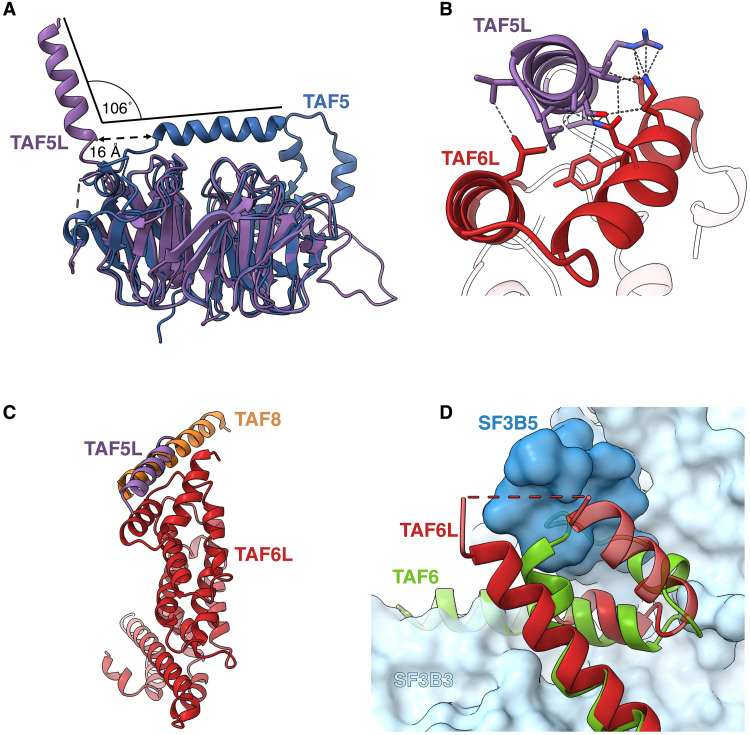
SAGA-specific adaptations enable SPL incorporation. (**A**) Comparison of human TAF5 [in TFIID, blue; Protein Data Bank (PDB): 7EGE] and TAF5L (in SAGA, violet). The TAF5L helix interacting with TAF6L HEAT repeat domain in SAGA is displaced compared to its counterpart in TAF5. (**B**) The same TAF5L helix forms a three-helix bundle with the HEAT repeat domain of TAF6L (red) in SAGA. The contacts are shown with dotted lines. (**C**) TAF8 (orange) in TFIID (PDB: 7EGE) interacts similarly with TAF6 as TAF5L (violet) with TAF6L (red) in SAGA. TAF6 HEAT repeats were aligned on TAF6L HEAT. Only TAF6L repeats are shown. (**D**) The last HEAT repeat of TAF6L (red) deviates from the one of TAF6 (green; PDB: 7EGE) to allow the incorporation of the splicing module (SF3B3, light blue surface; SF3B5, dark blue surface) into SAGA. The last HEAT repeat of TAF6 clashes with both SF3B3 and SF3B5.

The answer lies in gene duplication events, creating specialized genes for SAGA rather than genes shared with other complexes as is the case in yeast. The most substantial difference in sequence alignment between TAF6 and TAF6L lies in the linker that connects the histone fold with the HEAT repeat (fig. S5A). En route, this linker complements the first of the TAF5/TAF5L WD “propeller” blades with two β strands. In yeast and in human TAF6, this linker then continues with ~60 additional amino acids before forming the first HEAT repeat. In the SAGA-specialized TAF6L, only six residues separate the end of the β strands and the base of the first HEAT repeats helix (fig. S5B). Hence, the interaction of the linker with the TAF5L propeller forms an anchor point that strongly constrains the position of the TAF6L HEAT repeats. The second important adjustment occurs in the TAF5L helix that leads into the first WD propeller blade. In human TAF5L, this helix is shifted by 12 Å and tilted by 92° relative to yeast Taf5, and by 16 Å and 106° relative to human TAF5 (Fig. 5A) (*20*). This is due to the fact that this helix is connected to the first propeller blade via a much shorter linker in the TAF5L (five residues compared to 12 in both yeast and human TAF5). As a result, this helix in human SAGA runs antiparallel to the first HEAT repeat, with which it forms a three-helix bundle that has the most important contribution to the positioning and stability of the TAF6L HEAT repeat domains (Fig. 5B). Notably, in human TFIID, at the same position with respect to the TAF6 HEAT repeats in lobe C, we find a helix attributed to subunit TAF8 that runs parallel to the repeats and forms a three-helix bundle with the first repeat, very similar to the TAF5L helix (Fig. 5C). Thus, a common solution to the requirement of positioning the TAF6L/TAF6 HEAT repeats is applied both by SAGA and TFIID albeit with different subunits, namely, TAF5L and TAF8, respectively.

In addition to the long linker connecting the histone fold and HEAT repeats, the main structural difference between TAF6 and TAF6L appears in the last HEAT repeat, which assumes a very different angle in TAF6 and ends with a much longer helix. Superposing the TAF6 and TAF6L HEAT repeats shows that the TAF6 repeats would clash with SF3B3 (Fig. 5D), and the multitude of interactions formed by the tip of the last helix in TAF6L and SPL (Fig. 4F) would not be reproduced by the completely different sequence in TAF6. Hence, gene duplications that created proteins specialized for a role in SAGA were crucial for the structural rearrangement that is required for adding the splicing module and, probably, augmenting HAT autonomy.

## DISCUSSION

We present in this paper an example for an isolation based on small compound affinity ligand yielding an intact, active, and highly pure low-abundance multiprotein complex suitable for high-resolution structure determination. We could use our SAGA isolation method with two different cells lines, namely, K562 and HeLa S3, demonstrating the applicability of this concept for different sources. We further show the utility of this method and the quality of the sample by generating the highest resolution structure ever obtained of the human SAGA complex. In addition to the choice of the affinity ligand, we ascribe this success to two innovations we introduced, namely, a long linker to conjugate the affinity ligand to desthiobiotin and the use of PEG precipitation to clear the nuclear extract and concentrate it.

The structure of full GCN5/PCAF, let alone the holo-HAT module of SAGA/ATAC, has not been elucidated so far, and therefore, it is unknown where the bromodomain target of GSK4027 is situated within this module. We can only speculate that the affinity ligand with the long linker allows reaching the bromodomain without SAGA/ATAC clashing with the solid matrix. The bromodomain of GCN5/PCAF binds acetylated lysines on the N-terminal tail of histone H3, which is a long, flexible thread of roughly 40 amino acids. It is therefore possible that the bromodomain is not located on the surface of the HAT module but rather situated in a cavity where it is still accessible to the flexible H3 tail. Such a cavity would necessitate a long linker for GSK4027 to infiltrate inside without bringing SAGA/ATAC too close to the matrix.

The possibility to efficiently isolate intact and highly pure SAGA from practically any cell type leads us to suggest that purification of other coactivator complex is feasible provided that a suitable affinity ligand is available. Isolation of these complexes from different organisms or stages of development, from various organs, or from different pathologies could be crucial for elucidating the interactome of coactivators and their place in the pathways that lead to disease and differentiation. For the specific case of SAGA, which we addressed here, we note for example that spinocerebellar ataxia 7 is an adult-onset neurodegenerative disorder caused by the expansion of a CAG repeat sequence located within the coding region for SAGA DUB subunit ATXN7. The underlying molecular mechanism for the disease is not fully elucidated (*21*). Isolating SAGA from cells of patients (or animal models) at different ages and measuring its deubiquitination activity could shed new light on the physiopathology of this disease. Another instance is presented by regulatory T cells that attenuate the immune response, where FOXP3 is a master regulator of multiple pathways crucial for their function. SAGA plays a critical role in controlling FOXP3, and therefore, understanding the SAGA-FOXP3 interaction in these cells could help to better manage autoimmunity or cancer (*22*).

Our work reveals the high-resolution structures of the 150-kDa SPL on SAGA and the TAF6L HEAT repeats, as well as the molecular details of their incorporation into SAGA. Possibly, the very gentle nature of our purification scheme allowed us to determine maps with overall higher resolution and, in particular, better description of this peripheral region of SAGA. We could therefore compare in detail the interaction of SPL with TAF6L HEAT repeats in SAGA to their docking on SF3B1 in SF3B. Although both TAF6L and SF3B1 associate with the same face of SPL, the interaction networks are very different, apart from the last helix in both proteins that is positioned in the same place with respect to SPL. The last helices have very similar sequences at their last two turns and contribute substantially to the contact with SF3B3 and SF3B5.

At least in the case of TAF6, and possibly also of TAF5, the most important structural differences between the SAGA-specialized protein (TAF6L) and the canonical versions in TFIID or in yeast are directly related to the structural rearrangements required to accommodate the splicing module. These differences include a linker, shorter in TAF6L by ~50 amino acids compared to TAF6, that constrains the position of the TAF6 HEAT repeats, a shorter linker in TAF5L that positions a helix to form a helix bundle with the first HEAT repeat, and completely different last two helices of the TAF6L HEAT repeats where the TAF6 version would clash into the SPL and would fail to present important residues for binding the SPL. This suggests that, to a considerable extent, the gene duplication events in metazoans occurred to enable the incorporation of the splicing module, which is the most prominent addition to metazoan SAGA compared to yeast. This fact suggests that the role of the splicing module in vivo is of considerable importance, although elusive. Our work proposes where mutations can be introduced to uncouple SPL from the HEAT repeats and SAGA so that its role could be studied. Eliminating the last two HEAT repeats of TAF6L or possibly even just the last helix should considerably undermine the SAGA contact with SPL.

We find that the buried surface in the SAGA interaction with SPL is only half that in the SF3B counterpart. The known correlation between buried surface and binding affinity (*23*) clearly suggests that SPL is associated much stronger with SF3B than with SAGA. Substantially contributing to this firmer interaction is the 30–amino acid linker in SF3B1, without any parallel in SAGA, which is inserted deeply into the cleft between SF3B3 and SF3B5 and forms a very extensive network of contacts with both subunits. A protein quantification study indicates that SAGA proteins (SUPT20H or SUPT7L), splicing module proteins (SF3B3 and SF3B5), and the U2snRNP protein SF3B1 occur with similar abundance in K562 cells (*24*). These data therefore suggest that at least part of the U2snRNP population may lack the splicing module and needs to “rob” it from SAGA where SF3B3 and SF3B5 are weaker bound. Thus, we propose that SAGA may serve as a relay of splicing factors that transfers SPL for assembly into the prespliceosome when SF3B1 (or the entire U2snRNP) is in the proximity of chromatin-bound SAGA during transcription-coupled splicing. Findings in *Saccharomyces cerevisiae* seem to support this idea. The yeast SAGA physically binds the U2snRNP adenosine triphosphatase PRP5 and modulates its activity in pre-mRNA branch-point proofreading (*25*). This activity requires an interplay between SF3B1 and PRP5 (*26*). We further suggest that the role of SAGA in relaying SPL to SF3B/U2snRNP could facilitate synchronization between transcription initiation and splicing. It could also limit accumulation of functional U2snRNP in the vicinity of mRNA, thus perhaps preventing possible splicing errors.

We acknowledge limitations of this study. Our structure suggests that the most important differences between the SAGA paralogs of TAF5 and TAF6 and the canonical versions in TFIID or in yeast are directly related to the incorporation of SPL. This points to an important role of this module. However, our work did not include any cellular assays to support the structural findings or to uncover this enigmatic role of SPL. Our structure should serve as a framework to guide such assays in the future.

## MATERIALS AND METHODS

### Synthesis of the affinity ligands

Unless otherwise stated, all chemical reagents were purchased from abcr GmbH (Karlsruhe, Germany) and used without purification. When required, solvents were dried just before use. Thin-layer chromatography (TLC) was performed on precoated plates (0.25-mm silica gel 60, F_254_, Merck, Darmstadt, Germany). Products were purified by flash chromatography over silica gel (silica gel 60, 40 to 63 μm, Merck, Darmstadt, Germany). Nuclear magnetic resonance (NMR) spectra were recorded on a Bruker 400 MHz Avance III or Bruker 500 MHz Avance III instrument. ^1^H- and ^13^C-NMR chemical shifts δ are reported in parts per million (ppm) relative to their standard reference (^1^H: CHCl_3_ at 7.26 ppm, CD_2_HOD at 3.31 ppm; ^13^C: CDCl_3_ at 77.0 ppm, CD_3_OD at 49.0 ppm). Splitting patterns are assigned s = singlet, d = doublet, t = triplet, q = quartet, quin = quintet, and br. = broad signal. Infrared (IR) spectra were recorded on a Fourier transform infrared (FTIR) Nicolet 380 spectrometer in the attenuated total reflectance (ATR) mode, and absorption values ν are in wave numbers (in reciprocal centimeter). MS were recorded on an Agilent Technologies 6520 Accurate Mass QToF instrument, using electrospray ionization (ESI) mode. Mass data are reported in mass units (mass/charge ratio).

#### tert-Butyl (3R)-3-(pyridine-2-carbonylamino)piperidine-1-carboxylate (2)

*N*-Ethyl-*N’*-(3-dimethylaminopropyl)carbodimide hydrochloride (EDC.HCl) (359 mg, 1.87 mmol) and 4-dimethylaminopyridine (4-DMAP) (15 mg, 0.12 mmol) were added to a solution of (*R*)-3-amino-1-*N*-Boc-piperidine (250 mg, 1.25 mmol) and 2-picolinic acid (189 mg, 1.53 mmol) in anhydrous CH_2_Cl_2_ (20 ml) under inert atmosphere. The reaction mixture was stirred at room temperature (RT) until the starting piperidine was no more detected by TLC (~2.5 hours). Then, it was washed with saturated aqueous NaHCO_3_ and brine. The organic layer was dried over MgSO_4_, filtered, and reduced under vacuum. The crude residue was purified by flash chromatography on silica gel (PE/Et_2_O: 20/80) to yield compound 1 (374 mmol, 98%) as a white powder. Analyses were consistent with the literature data.

#### tert-Butyl(3R,5R)-3-(4-(methoxycarbonyl)phenyl)-5-(picolinamido)piperidine-1-carboxylate (3)

Compound 3 was obtained from compound 2 according to the protocol described by Van Steijvoort *et al.* (*13*).

#### Methyl 4-((3R,5R)-5-(picolinamido)piperidin-3-yl)benzoate (4)

Compound 3 (1.231 g, 2.80 mmol) was stirred in CH_2_Cl_2_/trifluoroacetic acid 9:1 (25 ml) at RT for 2 hours. Volatile was removed under vacuum, the residue was stirred in CH_2_Cl_2_/H_2_O 1:1 (20 ml), and pH was set at 12 with 1 N NaOH. The organic layer was separated, and the aqueous layer was extracted twice with CH_2_Cl_2_. The combined organic layers were dried over MgSO_4_, filtered, and reduced under vacuum to yield compound 4 (0.885 g, 92%) as a slightly yellow powder. Analytical data: ^1^H-NMR (CDCl_3_, 500 MHz) δ 8.49 (dd, *J* = 4.8, 0.8 Hz, 1H), 8.16 (d, *J* = 7.8 Hz, 1H), 7.94 (m, 3H), 7.80 (td, *J* = 7.7, 1.7 Hz, 1H), 7.38 (ddd, *J* = 7.6, 4.7, 1.2 Hz, 1H), 7.26 (d, *J* = 8.3 Hz, 2H), 4.25–4.07 (m, 1H), 3.86 (s, 3H), 3.42 (m, 1H), 3.17 (m, 1H), 2.90 (m, 1H), 2.62 (dd, *J* = 12.6, 11.2 Hz, 1H), 2.53 (dd, *J* = 12.2, 10.8 Hz, 1H), 2.35 (m, 1H), 1.68 (q, *J* = 12.1 Hz, 1H); ^13^C-NMR (CDCl_3_, 101 MHz) δ 166.79, 163.64, 149.64, 148.17, 147.87, 137.27, 129.76 (2C), 128.41, 126.94 (2C), 126.10, 122.10, 52.43, 51.9, 51.11, 47.45, 43.83, 38.11; High Resolution Mass Spectrometry (HRMS) (ESI) for C_19_H_22_N_3_O_3_ [M + H]^+^, calculated 340.4025, found 340.4032.

#### Methyl 4-((3R,5R)-1-methyl-5-(picolinamido)piperidin-3-yl)benzoate (5)

Formaldehyde (350 μl, 37%) was added to a stirred mixture of compound 4 (990 mg, 2.91 mmol) and acetic acid (35 μl) in MeOH (20 ml) at RT. Then, NaBH(OAc)_3_ (1.33 g, 6.27 mmol) was added portion wise over a 20-min period, and the mixture was stirred for 1 hour before the solvent was removed under vacuum. The crude residue was suspended in CHCl_3_ and washed with water at pH 12. The aqueous layer was washed three times with CHCl_3_, and the organic layers were combined together, dried over MgSO_4_, and reduced under vacuum to yield analytically pure compound 5 (943 mg, 92%) as a white powder. Analytical data: *R*_f_ 0.5 (CH_2_Cl_2_/MeOH 9:1); ^1^H-NMR (400 MHz, CDCl_3_) δ 8.53 (ddd, *J* = 4.8, 1.7, 0.9 Hz, 1H), 8.19 (dt, *J* = 7.8, 1.1 Hz, 1H), 8.01–7.93 (m, 3H), 7.85 (td, *J* = 7.7, 1.7 Hz, 1H), 7.42 (ddd, *J* = 7.6, 4.8, 1.2 Hz, 1H), 7.36–7.28 (m, 2H), 4.36 (m, 1H), 3.90 (s, 3H), 3.25 (d, *J* = 13.0 Hz, 1H), 3.12 (t, *J* = 14.6 Hz, 1H), 2.99 (d, *J* = 11.3 Hz, 1H), 2.38 (s, 3H), 2.32 (d, *J* = 15.8 Hz, 1H), 2.07 (t, *J* = 11.2 Hz, 1H), 1.97 (m, 1H), 1.57 (m, 1H); ^13^C-NMR (CDCl_3_, 101 MHz) δ 166.92, 163.68, 149.77, 148.24, 147.95, 137.438, 129.84 (2C), 128.58, 127.17 (2C), 126.18, 122.22, 61.68, 60.25, 52.00, 46.51, 45.93, 41.49, 37.21; HRMS (ESI) for C_20_H_24_N_3_O_3_ [M + H]^+^, calculated 353.4209, found 353.4212.

#### Ethyl 4-((3R,5R)-5-amino-1-methylpiperidin-3-yl)benzoate (6)

Picolinamide 5 (100 mg, 0.28 mmol) was stirred in refluxing HCl 12 N for 20 hours. The reaction mixture was reduced under vacuum, and the residue was coevaporated twice with EtOH. Then, it was stirred with acetyl chloride (0.1 ml) in refluxing EtOH (4 ml) for 3 hours. Volatile was removed, and the dry residue was suspended in AcOEt and washed with NaOH 2 N. The aqueous layer was extracted twice with AcOEt, and the combined organic layers were dried over MgSO_4_ to yield an orange oil that was purified by flash chromatography over silica gel (CHCl_3_/MeOH/H_2_O 9:1:0 to 10:6:1) to afford compound 6 (49 mg, 66%). Analytical data: *R*_f_ 0.2 (CH_2_Cl_2_/MeOH 9:1); ^1^H-NMR (400 MHz, CD_3_OD) δ 7.98 (d, *J* = 8.4 Hz, 2H), 7.39 (d, *J* = 8.4 Hz, 2H), 4.36 (q, *J* = 7.1 Hz, 2H), 3.13–2.90 (m, 4H), 2.38 (s, 3H), 2.18–2.08 (m, 1H), 2.05 (t, *J* = 11.9 Hz, 1H), 1.88 (t, *J* = 11.9 Hz, 1H), 1.41 (m, 1H), 1.40 (t, *J* = 7.1 Hz, 3H); ^13^C-NMR (101 MHz, CD_3_OD) δ 167.91, 149.74, 130.82, 130.19, 128.42, 63.13, 62.35, 62.07, 46.12, 42.70, 40.10, 14.60; HRMS (ESI) for C_15_H_23_N_2_O_2_^+^ [M + H]^+^, calculated 263.1754, found 263.1766.

#### Ethyl 4-((3R,5R)-5-((5-bromo-1-methyl-6-oxo-1,6-dihydropyridazin-4-yl)amino)-1-methylpiperidin-3-yl) benzoate (7)

A solution of 1,8-Diazabicyclo (5.4.0)undec-7-ene (DBU) (68 μl, 0.46 mmol), 4,5-dibromo-2-methylpyridazin-3(2H)-one (123 mg, 0.42 mmol), and compound 6 (120 mg, 0.42 mmol) in dimethylacetamide (1 ml) was stirred at 120°C overnight in an airtight sealed tube. The reaction mixture was then cooled to RT, CHCl_3_ (10 ml) was added, and the mixture was washed with 1 N NaOH (4 × 5 ml). The organic layer was dried over MgSO_4_, filtered, and reduced under vacuum. The crude residue was purified by flash chromatography over silica gel (AcOEt/EtOH 9:1) to afford compound 7 (116 mg, 57%). Analytical data: *R*_f_ 0.25 (AcOEt/EtOH 9:1). Analyses were consistent with the literature data (*13*).

#### 4-((3R,5R)-5-((5-Bromo-1-methyl-6-oxo-1,6-dihydropyridazin-4-yl)amino)-1-methylpiperidin-3-yl)benzoic acid (8)

Compound 8 was obtained from compound 7 following the procedure described by Bassi *et al.* (*12*).

#### N-(2-(2-(2-Azidoethoxy)ethoxy)ethyl)-6-((4R,5S)-5-methyl-2-oxoimidazolidin-4-yl)hexanamide (9a)

Desthiobiotin (100 mg, 0.47 mmol), 2-(2-(2-azidoethoxy)ethoxy)ethan-1-amine (*27*) (120 mg, 0.69 mmol), Et_3_N (200 μl, 1.43 mmol), and HOBt (63 mg, 0.57 mmol) were mixed in CH_2_Cl_2_ (1 ml) before EDC.HCl (118 mg, 0.62 mmol) was added. The reaction mixture was stirred at RT for 24 hours before it was washed with 2% HCl, water, and brine. The organic layer was dried over MgSO_4_, filtered, and reduced under vacuum. The crude residue was purified by flash chromatography over silica gel (CHCl_3_/MeOH 100:0 to 98:2) to afford compound 9a (104 mg, 61%). Analytical data: *R*_f_ 0.8 (CHCl_3_/MeOH 8:2); ^1^H-NMR (CDCl_3_, 400 MHz) δ 6.47 (br., 1H), 5.69 (br., 1H), 4.94 (br., 1H), 3.81 (dq, *J* = 7.8, 6.4 Hz, 1H), 3.71–3.60 (m, 7H), 3.56 (dd, *J* = 5.6, 4.5 Hz, 2H), 3.44 (t, *J* = 4.9 Hz, 2H), 3.38 (dd, *J* = 5.5, 4.4 Hz, 2H), 2.17 (t, *J* = 7.4 Hz, 2H), 1.65 (p, *J* = 7.1 Hz, 2H), 1.52–1.23 (m, 6H), 1.11 (d, *J* = 6.5 Hz, 3H); ^13^C-NMR (CDCl_3_, 101 MHz) δ 173.10, 163.74, 70.44, 70.13, 70.07, 70.04, 56.08, 51.42, 50.65, 39.11, 35.95, 29.47, 28.63, 25.90, 25.21, 15.77; FTIR (thin film) ν 1650, 1697, 2107, 2863, 2933, 3270; HRMS (ESI) for C_16_H_31_N_6_O_4_^+^ [M + H]^+^, calculated 371.2401, found 371.2392.

#### N-(17-Azido-3,6,9,12,15-pentaoxaheptadecyl)-6-((4R,5S)-5-methyl-2-oxoimidazolidin-4-yl)hexanamide (9b)

Compound 9b (198 mg, 85%) was obtained from desthiobiotin and 17-azido-3,6,9,12,15-pentaoxaheptadecan-1-amine (*28*) following the same protocol as for compound 9a. Analytical data: *R*_f_ 0.65 (CHCl_3_/MeOH 8:2); ^1^H-NMR (CDCl_3_, 400 MHz) δ 6.55 (t, *J* = 5.6 Hz, 1H), 5.63 (s, 1H), 4.86 (s, 1H), 3.84–3.77 (m, 1H), 3.67 (t, *J* = 4.9 Hz, 2H), 3.67–3.60 (m, 17H), 3.55 (dd, *J* = 5.6, 4.5 Hz, 2H), 3.42 (d, *J* = 5.4 Hz, 2H), 3.37 (d, *J* = 5.4 Hz, 2H), 2.18 (t, *J* = 7.4 Hz, 2H), 1.52–1.23 (m, 6H), 1.10 (d, *J* = 6.5 Hz, 3H); ^13^C-NMR (CDCl_3_, 101 MHz) δ 174.0, 163.6, 70.7–69.9 (br.), 56.01, 51.35, 50.64, 39.11, 35.92, 29.44, 28.61, 25.88, 25.16, 15.73; FTIR (thin film) ν 1697, 2100, 2863, 2930, 3292; HRMS (ESI) for C_22_H_43_N_6_O_7_^+^ [M + H]^+^, calculated 503.3188, found 503.3172.

#### N-(2-(2-(2-Aminoethoxy)ethoxy)ethyl)-6-((4R,5S)-5-methyl-2-oxoimidazolidin-4-yl)hexanamide (10a)

Azide 9a (403 mg, 1.1 mmol) and 10% Pd/C (75 mg) in EtOH (10 ml) were vigorously stirred under a H_2_ atmosphere for 2 hours. The catalyst was filtered off, and the volatile was removed under vacuum to yield compound 10a (370 mg, 99%). Analytical data: *R*_f_ 0.2 (CH_2_Cl_2_/MeOH 8:2); ^1^H-NMR (CD_3_OD, 400 MHz) δ 3.91–3.75 (m, 1H), 3.75–3.66 (m, 1H), 3.63 (m, 4H), 3.55 (t, *J* = 5.6 Hz, 3H), 3.36 (t, *J* = 5.6 Hz, 2H), 2.82 (t, *J* = 5.3 Hz, 2H), 2.21 (t, *J* = 7.5 Hz, 2H), 1.65–1.57 (m, 4H), 1.53–1.42 (m, 2H), 1.40–1.29 (m, 2H), 1.11 (d, *J* = 6.4 Hz, 3H); ^13^C-NMR (CD_3_OD, 101 MHz) δ 176.28, 164.62, 72.81, 71.33, 71.26, 70.62, 57.39, 52.70, 41.90, 40.27, 36.90, 30.74, 30.21, 27.15, 26.81, 15.62; FTIR (thin film) ν 1691, 2864, 2933, 3269; HRMS (ESI) for C_16_H_33_N_4_O_4_^+^ [M + H]^+^, calculated 345.2496, found 345.2513.

#### N-(17-Amino-3,6,9,12,15-pentaoxaheptadecyl)-6-((4R,5S)-5-methyl-2-oxoimidazolidin-4-yl)hexanamide (10b)

Compound 10b (210 mg, 99%) was obtained from compound 9b following the same protocol as for compound 10a. Analytical data: *R*_f_ 0.2 (CH_2_Cl_2_/MeOH 8:2); ^1^H-NMR (CD_3_OD, 400 MHz) δ 3.86–3.79 (m, 1H), 3.78–3.73 (m, 2H), 3.73–3.59 (m, 17H), 3.55 (t, *J* = 5.6 Hz, 2H), 3.36 (t, *J* = 5.6 Hz, 2H), 3.20–3.09 (m, 2H), 2.22 (t, *J* = 7.4 Hz, 3H), 1.64 (quin, *J* = 7.3 Hz, 2H), 1.56–1.21 (m, 6H), 1.11 (d, *J* = 6.4 Hz, 3H); ^13^C-NMR (CDCl_3_, 101 MHz) δ 176.3, 166.1, 79.59, 71.5 (br.), 71.42, 71.31, 71.26, 71.23, 71.14, 71.00, 70.65, 67.87, 57.36, 36.90, 30.73, 30.19, 27.13, 26.79, 15.64; FTIR (thin film) ν 1647, 1678, 2869, 3269; HRMS (ESI) for C_22_H_45_N_4_O_7_^+^ [M + H]^+^, calculated 477.3283, found 477.3271.

#### 4-((3R,5R)-5-((5-Bromo-1-methyl-6-oxo-1,6-dihydropyridazin-4-yl)amino)-1-methylpiperidin-3-yl)-N-(2-(2-(2-(6-((4R,5S)-5-methyl-2-oxoimidazolidin-4-yl)hexanamido)ethoxy)ethoxy)ethyl)benzamide (1a)

A mixture of compound 8 (20 mg, 47.6 μmol), compound 10a (24 mg, 69.6 μmol), EDC.HCl (37 mg, 193.6 μmol), HOBt (9 mg, 66.6 μmol), and Et_3_N (80 μl, 574 μmol) was stirred in anhydrous CH_2_Cl_2_ (4 ml) overnight at RT. The crude reaction mixture was washed with 1 N NaOH and brine, and the organic layer was dried over Na_2_SO_4_. The solvent was removed under vacuum, and the residue was purified by flash chromatography over silica gel (CHCl_3_/MeOH 100:0 to 80:20) to afford compound 9a (33 mg, 93%). Analytical data: *R*_f_ 0.6 (CHCl_3_/MeOH 8:2); ^1^H-NMR (CDCl_3_, 400 MHz) δ 7.77 (d, *J* = 8.3 Hz, 2H), 7.55 (s, 1H), 7.28 (d, *J* = 8.3 Hz, 2H), 6.98 (t, *J* = 5.5 Hz, 1H), 6.52 (t, *J* = 5.6 Hz, 1H), 5.78 (s, 1H), 4.86 (s, 1H), 4.65 (d, *J* = 8.7 Hz, 1H), 3.86–3.79 (m, 1H), 3.79 (q, *J* = 7.7 Hz, 1H), 3.74 (s, 3H), 3.69–3.59 (m, 7H), 3.54 (t, *J* = 5.3 Hz, 2H), 3.42–3.38 (m, 2H), 3.14 (d, *J* = 11.0 Hz, 1H), 3.04 (tt, *J* = 11.9, 3.7 Hz, 1H), 2.94 (d, *J* = 10.9 Hz, 1H), 2.36 (s, 3H), 2.28 (d, *J* = 12.4 Hz, 1H), 2.15 (t, *J* = 7.3 Hz, 2H), 2.05 (t, *J* = 11.2 Hz, 1H), 1.92 (t, *J* = 10.6 Hz, 1H), 1.62 (quin, *J* = 7.1 Hz, 2H), 1.54–1.22 (m, 7H), 1.09 (d, *J* = 6.4 Hz, 3H); ^13^C-NMR (CDCl_3_, 101 MHz) δ 173.10, 167.12, 163.68, 157.91, 145.76, 144.92, 133.11, 127.44, 127.21, 124.99, 100.05, 70.15, 70.12, 69.91, 69.81, 61.50, 61.04, 55.96, 51.38, 50.07, 45.84, 41.36, 40.34, 39.74, 39.08, 37.97, 35.90, 29.45, 28.53, 25.80, 25.16, 15.74; FTIR (thin film) ν 1604, 1693, 2930, 3292; HRMS (ESI) for C_34_H_52_BrN_8_O_6_^+^ [M + H]^+^, calculated 747.3188, found 747.3200.

#### 4-((3R,5R)-5-((5-Bromo-1-methyl-6-oxo-1,6-dihydropyridazin-4-yl)amino)-1-methylpiperidin-3-yl)-N-(24-((4R,5S)-5-methyl-2-oxoimidazolidin-4-yl)-19-oxo-3,6,9,12,15-pentaoxa-18-azatetracosyl)benzamide (1b)

Compound 1b (43 mg, 56%) was obtained from compounds 8 and 10b following the same protocol as for compound 1a. Analytical data: *R*_f_ 0.7 (CH_2_Cl_2_/MeOH 8:2); ^1^H-NMR (CDCl_3_, 400 MHz) δ 7.81 (d, *J* = 8.3 Hz, 2H), 7.56 (s, 1H), 7.28 (d, *J* = 12.5 Hz, 2H), 7.23 (t, *J* = 5.4 Hz, 1H), 6.67 (t, *J* = 5.6 Hz, 1H), 5.41 (s, 1H), 4.74 (s, 1H), 4.63 (d, *J* = 8.7 Hz, 1H), 3.92–3.79 (m, 1H), 3.81 (quin, *J* = 6.7 Hz, 1H), 3.75 (s, 3H), 3.68–3.57 (m, 21H), 3.52 (t, *J* = 5.3 Hz, 2H), 3.48–3.35 (m, 2H), 3.17 (d, *J* = 11.0 Hz, 1H), 3.11–3.02 (m, 1H), 2.98 (d, *J* = 12.4 Hz, 1H), 2.38 (s, 3H), 2.28 (d, *J* = 12.1 Hz, 1H), 2.18 (t, *J* = 7.4 Hz, 1H), 2.07 (t, *J* = 11.2 Hz, 1H), 1.95 (t, *J* = 10.8 Hz, 1H), 1.63 (quin, *J* = 7.1 Hz, 2H), 1.58–1.15 (m, 7H), 1.10 (d, *J* = 6.5 Hz, 3H); ^13^C-NMR (CDCl_3_, 101 MHz) δ 173.11, 167.07, 163.45, 157.91, 145.41, 144.91, 133.30, 127.60, 127.10, 124.99, 100.12, 70.44, 70.42 (br.), 70.10, 70.03, 69.94, 69.92, 61.48, 60.94, 55.97, 51.34, 50.00, 45.77, 41.31, 40.36, 39.79, 39.11, 37.98, 35.99, 29.64, 29.48, 28.76, 25.94, 25.28, 15.74; FTIR (thin film) ν 1605, 1689, 2865, 2929, 3292; HRMS (ESI) for C_40_H_64_BrN_8_O_9_^+^ [M + H]^+^, calculated 879.3974, found 879.3971.

### Cell preparation

K562 cells (from American Type Culture Collection, CCL-243) were cultured in RPMI supplemented with 10% calf serum and gentamycin (40 μg/ml), at 37°C in 5% CO_2_ and 80-rpm rotative incubator. The cells have been grown into 3 liters at 1.5 × 10^6^ C/ml, for a total of 4.5 × 10^9^ cells.

HeLa cells were cultured in minimum essential medium spinner modification (S-MEM) supplemented with 7% newborn calf serum, 2 mM glutamine, and gentamycin (40 μg/ml) at 37°C in 5% CO_2_. The cells have been grown into 4.5 liters at 1 × 10^6^ C/ml, for a total of 4.5 × 10^9^ cells.

### Purification of SAGA/ATAC complexes

Three or four and half–liter cultures were centrifuged (1000*g* for 10 min). All following steps were performed at 0° to 4°C. After centrifugation, the cell pellet was incubated 30 min on ice to equilibrate the transcription state, then was first washed with cold phosphate-buffered saline followed by a wash with K75 buffer [10 mM Hepes, pH 7.9; 75 mM KCl; 1.5 mM MgCl_2_; 1 mM dithiothreitol (DTT); pepstatin A (5 μg/ml); and E64 (3 μg/ml)]. The cell pellet was then resuspended in hypotonic buffer K0 [10 mM Hepes, pH 7.9; 1.5 mM MgCl_2_; 1 mM DTT; pepstaine A (5 μg/ml); E64 (3 μg/ml); and Roche protease inhibitor cocktail] and homogenized in a 100-ml B-Dounce homogenizer. Sucrose was added at a final concentration of 10% (w/v), and nuclei were pelleted for 28 min at 5000*g*. Nuclear pellets were washed with sucrose buffer [10 mM Hepes, pH 7.9; 10 mM KCl; 1.5 mM MgCl_2_; 10% sucrose (w/v); 1 mM DTT; pepstaine A (5 μg/ml); and E64 (3 μg/ml)]. Nuclear pellets were resuspended in no-salt buffer [20 mM Hepes, pH 7.9; 1.5 mM MgCl_2_; 25% glycerol (w/v); 2 mM DTT; pepstaine A (5 μg/ml); E64 (3 μg/ml); and Roche protease inhibitor cocktail] and homogenized in a 40-ml B-Dounce homogenizer. NaCl (0.3 M) were added before a 30-min incubation with rotation. The nuclear extract was cleared by centrifugation (30,000*g*, 30 min) and flash-frozen in liquid nitrogen.

PEG 20,000 (1.2%) and 5 mM MgCl_2_ were added to precipitate some remaining cellular organelles and membranes by centrifugation at 33,000*g* for 10 min. PEG 20,000 was then added at a 4.5% final concentration to precipitate large protein complexes with the same centrifugation step. The pellet was then resuspended in buffer A [20 mM Hepes, pH 8.0; 250 mM NaCl; 2 mM MgCl_2_; 10% sucrose (w/v); 0.5 mM DTT; pepstaine A (5 μg/ml); E64 (3 μg/ml); 0.05% Tween 20; and Roche protease inhibitor cocktail]. The sample was mixed with streptavidin sepharose high-performance beads (Cytiva) equilibrated in buffer A and incubated with rotation for 4 hours to remove endogenous biotinylated proteins. In parallel, 35 μl of streptavidin sepharose high-performance beads were incubated for 2 hours with 2.5 μl of the affinity ligand (10 mM stock solution), containing either the short (1a) or the long (1b) linker. The saturated beads were then washed twice with buffer A. The prepared sample and beads were mixed and incubated overnight. We deliberately used an amount of beads that would bind only 70% of the total mass of SAGA/ATAC in order to assure high purity of the sample. The beads were subjected to six washes: three with buffer B [20 mM Hepes, pH 8.0; 250 mM NaCl; 2 mM MgCl_2_; 15% sucrose (w/v); 0.5 mM DTT; pepstaine A (5 μg/ml); E64 (3 μg/ml); and 0.05% Tween 20], one with buffer C [20 mM Hepes, pH 8.0; 250 mM NaCl; 2 mM MgCl_2_; 7.5% sucrose (w/v); 5% trehalose; 2.5 μM β-mercaptoethanol (BME); and 0.0025% dodecyl-maltoside (DDM)], and two with buffer D (20 mM Hepes, pH 8.0; 250 mM NaCl; 2 mM MgCl_2_; 5% trehalose; 2.5 μM BME; and 0.002% DDM). The beads were finally eluted in buffer D without trehalose and containing 20 mM biotin. The eluate was used for both cryo-EM analysis and biochemical analysis.

We note that total signal derived from SAGA/ATAC subunits out of total PSM in the MS analysis is only 30% with the short linker, compared to 60% with the longer linker. Furthermore, the purification with the short linker–based conjugate produced overall 12 times less SAGA/ATAC than the purification with the longer version.

### Western blots

In Western blots for Fig. 2 and figs. S2 and S3, to allow comparison between the content of the nuclear extract to the sample following PEG precipitation, we diluted the sample after PEG precipitation by the ratio between the volumes of the extract and the sample (roughly 20). The primary antibodies used are against TADA2B (produced in-house, no. 3122), GCN5 (produced in-house, no. 2676), and histone H2B (Cell Signaling, 2934S).

### Cryo-EM sample preparation and data acquisition

The sample was freshly cross-linked with 0.15% glutaraldehyde (GA). Three microliters of the sample, concentrated at 0.15 mg/ml, was applied onto one side of a holey gold EM grid (UltrAuFoil R1.2/1.3 mesh) rendered hydrophilic by 90-s treatment in Fischione 1070 plasma cleaner operating at 34% power with a gas mixture of 80% argon and 20% oxygen. One microliter of the sample was added on the other side of the grid. The grid was blotted on the 1-μl side for 5 s and flash-frozen in liquid ethane using an EM GP2 Automatic Plunge Freezer at 6°C and 90% humidity.

### Data acquisition

The image dataset was acquired on a Titan Krios G4 microscope operating at 300 kV in nanoprobe mode using SerialEM for automated data collection. Movie frames were recorded on a Flacon 4i direct electron detector after a Selectris X energy filter using a 10-eV slit width in zero-loss mode. Images were acquired at nominal magnification of 165,000×, yielding a pixel size of 0.729 Å.

### Image processing

The image dataset was preprocessed using cryoSPARC (*29*). Particle coordinates were determined using crYOLO. Particle images were extracted using Relion 5 (*30*) with box size of 560 pixels rescaled to 256 pixels, generating a pixel size of 1.595 Å. The dataset was analyzed in Relion 5 and cryoSPARC according to standard protocols. Briefly, three rounds of reference free 2D classification of the individual particle images were performed in cryoSPARC to remove images corresponding to contaminating or damaged particles and ice contaminations. References (3D models) were generated by the ab initio 3D reconstruction program of cryoSPARC. These structures were then used as references for 3D classification jobs in cryoSPARC, and particles corresponding to high-resolution 3-D classes were selected and used for nonuniform refinement. The selected particles were re-extracted in Relion 5 with box size of 560 pixels rescaled to 360 pixels, generating a pixel size of 1.134 Å. These particles were refined in cryoSPARC and subjected to 3D classification in Relion 5 without alignment using various regularization parameter (*T*) values. Particles corresponding to high-resolution classes were used in the subsequent nonuniform refinement in cryoSPARC.

Focused refinements were carried out in cryoSPARC using masks covering the regions of interest created in ChimeraX (*31*). The number of ATAC particles was much smaller than SAGA, and most were removed by 2D classification.

### Model building

Protein Data Bank (PDB) structures 7KTS and 8H7G were used as starting models for building the atomic model. The model was refined in Phenix (*32*) by real-space refinement with secondary structure restraints and in Isolde. All display images were generated using ChimeraX.

### Reconstitution of histone octamers and preparation of nucleosome DNA

Octamers were reconstituted from individual *Xenopus laevis* (canonical) histones expressed as inclusion bodies according to the standard protocol (*33*, *34*). Widom-601 145–base pair (bp) DNA was produced using a plasmid harboring 16 copies of this sequence as described by Dyer *et al.* (*33*).

### Nucleosome reconstitution

Nucleosomes with 145-bp Widom-601 positioning sequence were prepared according to NEB Dilution Assembly Protocol (E5350) (https://international.neb.com/protocols/2012/06/02/dilution-assembly-protocol-e5350) with some modifications as follows: 2.75 μM 145-bp Widom-601 DNA was mixed with 2.75 μM canonical histone octamers in a solution containing 2 M NaCl, 1 mM EDTA, and 5 mM BME. The solution was incubated for 30 min at RT and then underwent serial dilutions down to 1.48, 1, 0.6, and 0.25 M NaCl with buffer LowSalt (10 mM Hepes-KOH, pH 8.0, and 2.5 mM BME). After each dilution, the solution was kept at RT for 30 min. To reduce the final NaCl concentration, nucleosomes were concentrated in 0.5 ml of 100-kDa cutoff Amicon up to 100 μl and then diluted five times with buffer LowSalt. This step was repeated one more time. Last, nucleosomes were concentrated to 3 to 4 μM and analyzed in a 5% native 0.2× tris-borate EDTA polyacrylamide gel to ascertain the quality of the sample and absence of free DNA.

### HAT assay

The reaction was performed in 20 mM Hepes (pH 7.5), 50 mM NaCl, 2 mM MgCl_2_, 10% glycerol (v/v), bovine serum albumin (BSA; 100 μg/ml), 2.5 nM BME, 50 μM acetyl-CoA, and 1 μM nucleosome. The reaction was started with the addition of 20 nM of SAGA/ATAC purified complexes. After 30 min of incubation at 37°C, the reaction was stopped by an addition of SDS loading buffer (Laemmli buffer) and heated at 95°C for 3 min. Proteins were then resolved by 15% SDS-PAGE and analyzed by Western blot. The primary antibody used was against acetylated lysine (Cell Signaling Technology, 9441S).

### Deubiquitination assay

To test purified SAGA DUB activity, Ub-AMC from ENZO (BML-SE211) was used. The reaction buffer is composed of 20 mM Hepes (pH 8.0), 150 mM potassium acetate, 5 mM MgCl_2_, 20% sucrose (w/v), 0.005% DDM, BSA (0.1 mg/ml), and 230 nM Ub-AMC. The reaction was initiated with the addition of 20 nM of SAGA and was performed in 96-well plate at RT in the dark. Measurements were done at 0, 5, 10, 15, 20, 25, 30, and 60 min in the PHERAstar^Plus^ plate reader with a wavelength excitation at 355 nm and emission at 460 nm. The emission intensity of Ub-AMC without enzyme was also measured as control.
