# Supplementary material for: Insights into the structure and evolution of the human SAGA complex by affinity-ligand purification
Source: Sci Adv. 2026 Mar 18;12(12):eaec8104. doi: 10.1126/sciadv.aec8104 (PMC12998509; doi:10.1126/sciadv.aec8104)
Supplement: Supplementary file 1 — Figs. S1 to S6 Table S1 [file sciadv.aec8104_sm.pdf]

Science Advances
AAAS

Supplementary Materials for

Insights into the structure and evolution of the human SAGA complex by affinity-ligand purification

Mylène Damilot *et al.*

Corresponding author: Luc Lebeau, llebeau@unistra.fr; Gabor Papai, papai@igbmc.fr; Adam Ben-Shem, adam@igbmc.fr

Sci. Adv. **12**, eaec8104 (2026)
DOI: 10.1126/sciadv.aec8104

This PDF file includes:

Figs. S1 to S6
Table S1

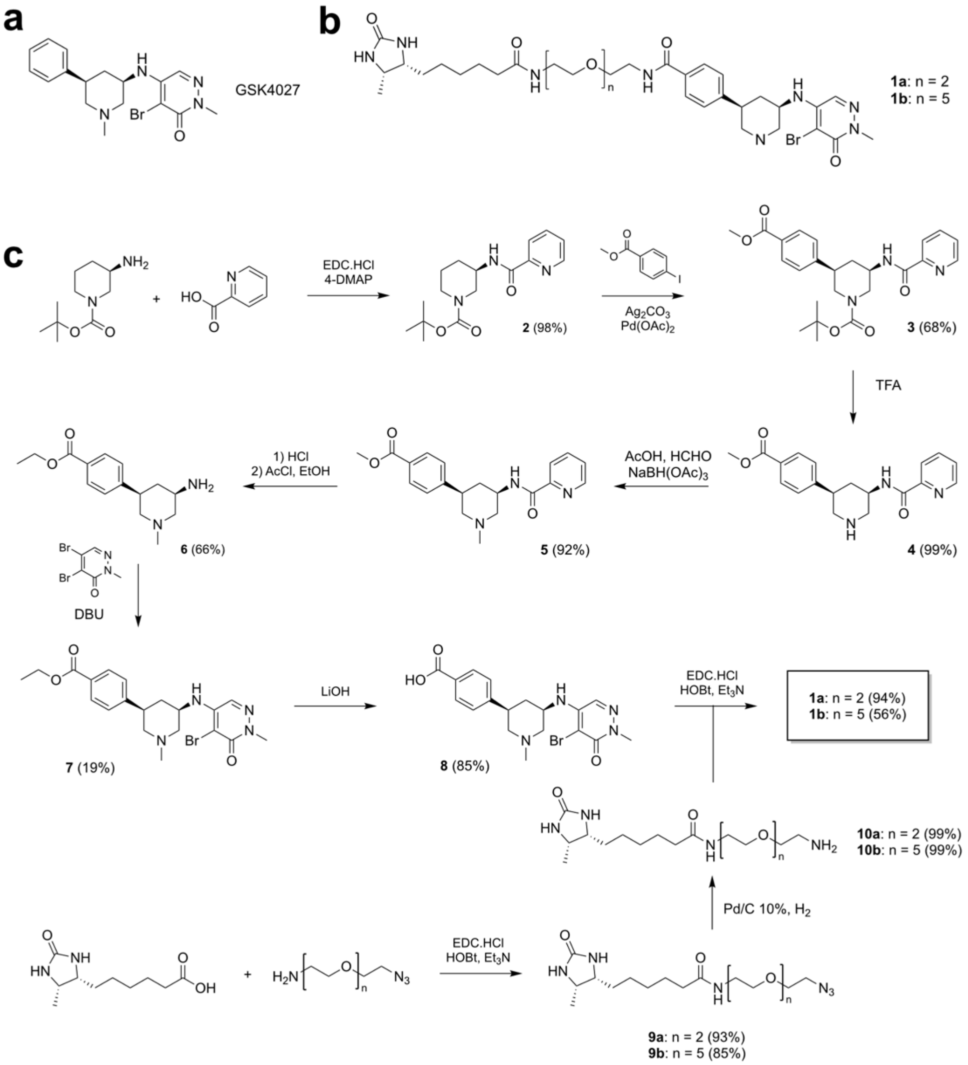

Supplemental Figure 1: Synthesis of the affinity-ligands.

a, structure of GSK4027. **b,** Structure of affinity ligands 1a and 1b. **c,** details of the synthetic route to prepare the compounds.

a

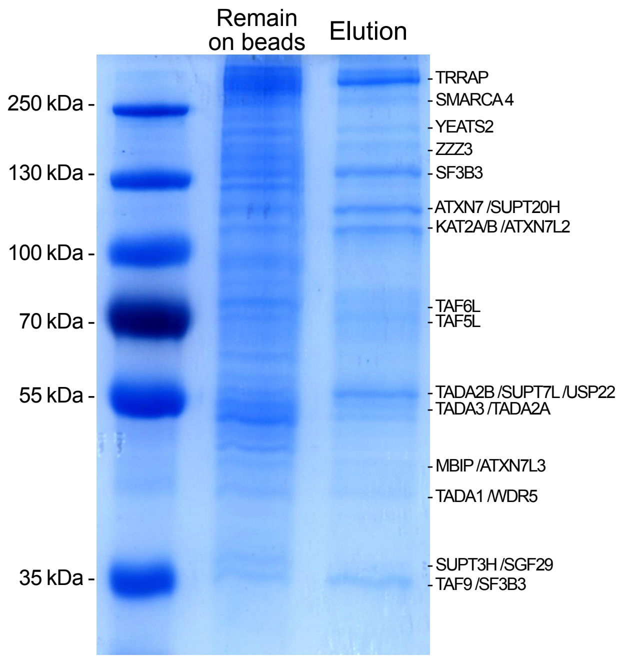

b

Real position in MS list	Description	NSAF (PSM/AA)	Normalization (SUPT20H)
1	TRRAP	0.91	2.2
2	SF3B3	0.97	2.4
3	BPTF	0.23	0.6
4	YEATS2	0.49	1.2
5	KAT2A	0.75	1.9
6	TAF6L	1.00	2.5
7	TADA3	1.14	2.8
8	SMARCA5	0.47	1.2
9	TAF5L	0.77	1.9
10	BRD3	0.62	1.5
11	ATXN7	0.42	1.0
12	TADA2B	0.88	2.2
13	KAT2B	0.44	1.1
14	TAF9B	1.42	3.5
15	BRD4	0.25	0.6
16	ZZZ3	0.36	0.9
17	SUPT20H	0.40	1.0
18	TAF9	1.17	2.9
19	DDX17	0.41	1.0
20	ATXN7L2	0.40	1.0
22	TADA1	0.79	1.9
28	SGF29	0.72	1.8
31	USP22	0.34	0.8
33	MBIP	0.51	1.3
36	KAT14	0.21	0.5
37	ATXN7L3	0.48	1.2
38	SUPT7L	0.39	1.0
44	ATXN7L1	0.17	0.4
46	TADA2A	0.31	0.8
59	SUPT3H	0.34	0.8
77	TAF10	0.39	1.0
79	SF3B5	0.93	2.3
80	WDR5	0.24	0.6
81	TAF12	0.48	1.2
138	ENY2	0.43	1.1
227	DR1	0.13	0.3

Supplemental Figure 2: Long linker purification on HeLa cells biochemical analysis

a, Colloidal Coomassie blue stained SDS-PAGE of the purified SAGA/ATAC complexes. **b,** Proteomic analysis of the purified SAGA and ATAC complexes. For each identified protein the table shows the NSAF (Normalized Spectral Abundance Factor) value calculated from the Peptide Spectrum Matches (PSM) divided by the number of amino acids, and the normalization of the NSAF value to SUPT20H as rough estimation of stoichiometry. SAGA subunits are colored purple, ATAC subunits are colored blue, purple bold represents the common subunits between SAGA and ATAC, and co-purified bromodomain containing proteins are in green.

a

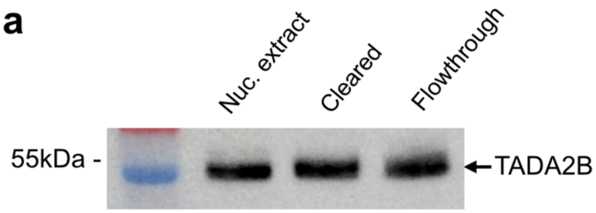

b

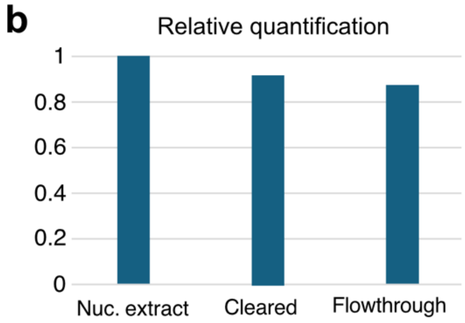

c

Real position in MS list	Description	NSAF (PSM/AA)	NSAF norm. to SUTP20H
1	TRRAP	1.09	1.5
2	SF3B3	1.17	1.6
**3**	**TADA3**	**1.89**	**2.5**
4	YEATS2	0.57	0.8
**5**	**KAT2A**	**0.93**	**1.3**
**6**	**KAT2B**	**0.88**	**1.2**
7	TAF6L	1.14	1.5
8	TAF5L	1.19	1.6
9	BPTF	0.22	0.3
10	ATXN7L2	0.85	1.1
11	SUPT20H	0.74	1.0
12	TADA2B	1.32	1.8
13	TAF9	2.01	2.7
14	SMARCA5	0.44	0.6
15	TAF9B	1.82	2.4
16	ZZZ3	0.47	0.6
17	ATXN7	0.43	0.6
18	DDX5	0.62	0.8
19	TADA1	1.11	1.5
20	DDX17	0.50	0.7
**26**	**SGF29**	**1.04**	**1.4**
32	SUPT7L	0.60	0.8
37	KAT14	0.29	0.4
39	SUPT3H	0.69	0.9
40	ATXN7L3	0.63	0.8
42	USP22	0.41	0.6
44	MBIP	0.60	0.8
49	TADA2A	0.43	0.6
64	ATXN7L1	0.18	0.2
79	TAF12	0.81	1.1
102	WDR5	0.29	0.4
109	SF3B5	1.08	1.5
140	TAF10	0.36	0.5
217	ENY2	0.49	0.7
189	DR1	0.20	0.3

d

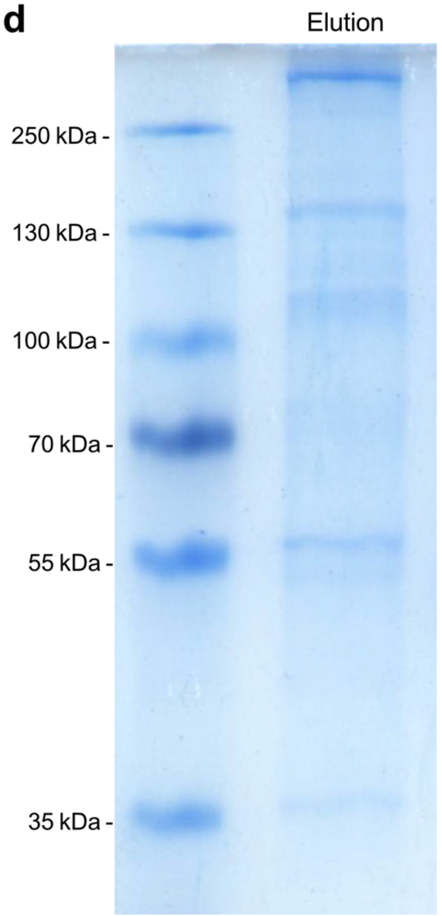

Supplemental Figure 3: Purification of SAGA/ATAC using affinity-ligand with short linker

a, Western blot analysis of different purification steps detecting the TADA2B subunit of SAGA. **b,** relative quantification of the detected bands in panel a. **c,** Proteomic analysis of the purified SAGA and ATAC complexes. For each identified protein the table shows the NSAF value calculated from the Peptide Spectrum Matches (PSM) divided by the number of amino acids, and the normalization of the NSAF value to SUPT20H as rough estimation of stoichiometry. SAGA subunits are colored purple, ATAC subunits are colored blue, purple bold represents the common subunits between SAGA and ATAC, and co-purified bromodomain containing proteins are in green. **d,** Colloidal Coomassie blue stained SDS-PAGE of the purified SAGA and ATAC complexes.

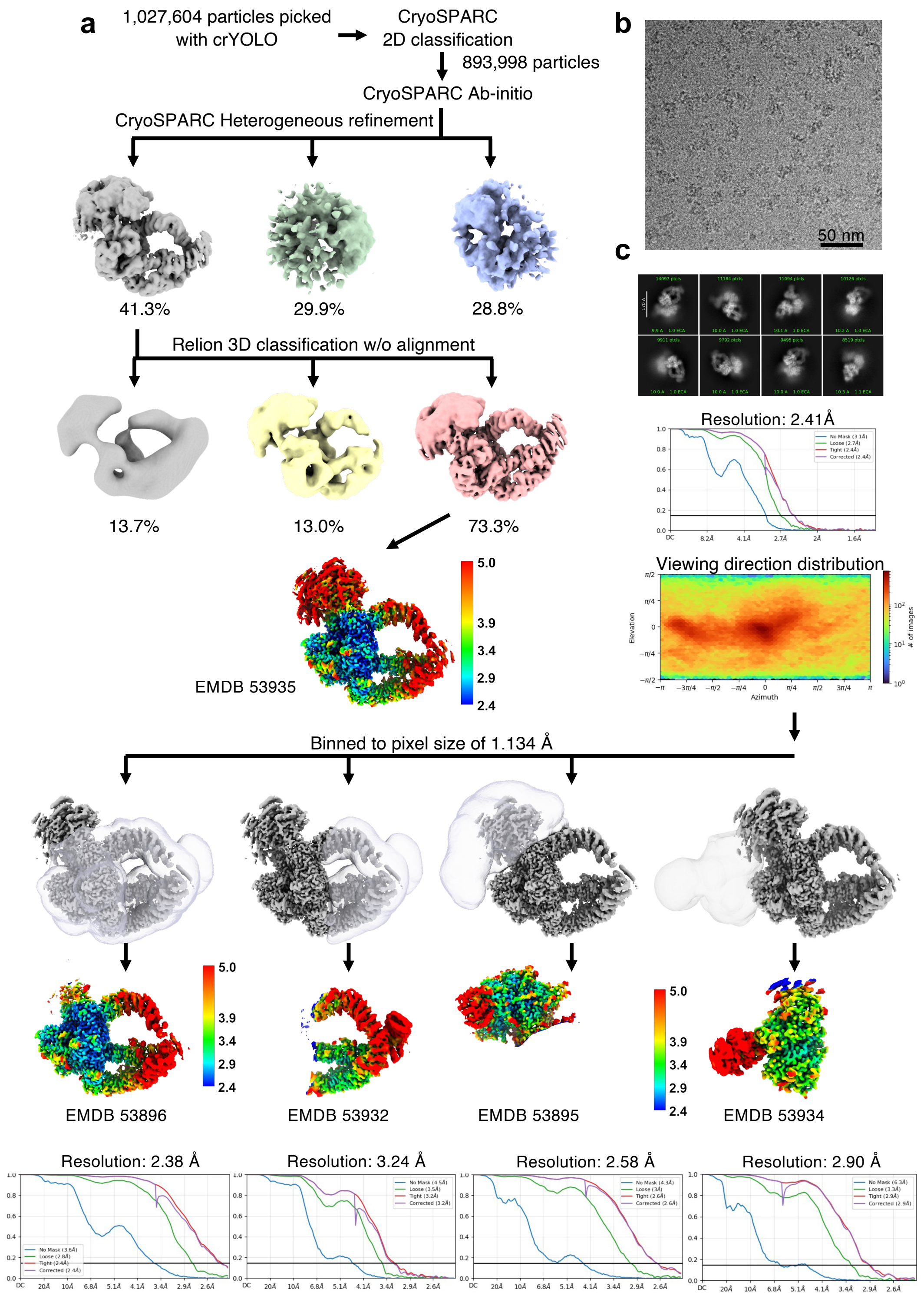

Supplementary Figure 4: Cryo-EM data analysis strategy and resolution assessment

a, Complete data processing scheme. Density map colored in rainbow represent the local resolution of the reconstructions. Hollow grey volumes represent the masks used for focused classification resolution in angstrom (Å) CryoSPARC v.4 were used to generate the 2.4 Å overall resolution map of human SAGA complex. RELION5 with Blush was used for classifications without alignment. **b,** Original micrograph. **c,** Two-dimensional class averages showing high-resolution structural features.

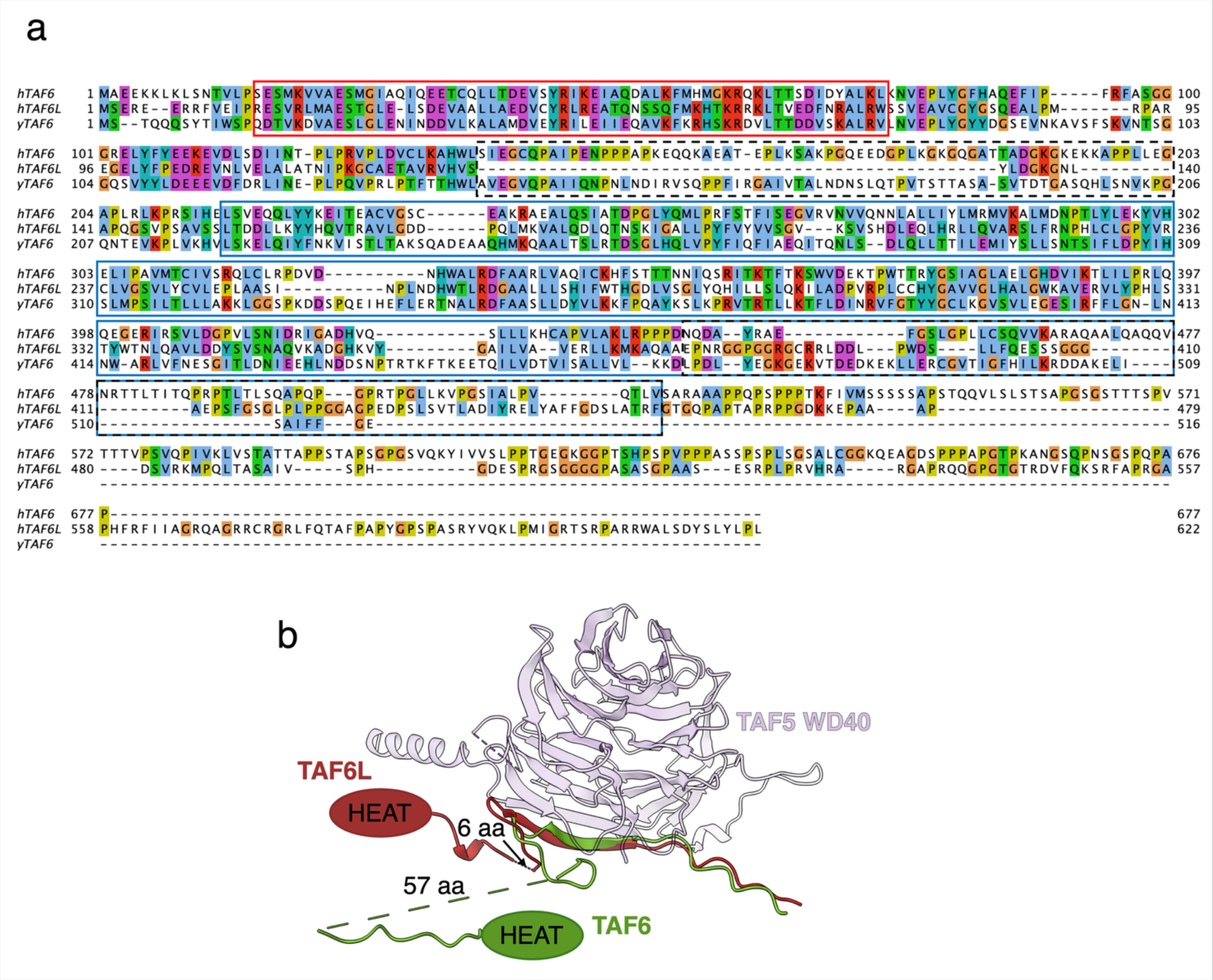

Supplementary Figure 5: Sequence alignment of TAF6 and TAF6L

a, Human TAF6, TAF6L and S. cerevisiae TAF6 were aligned using Clustal Omega. Boxes indicate structural features. Red: histone-fold domain; blue: HEAT repeat domain; dashed black: linker connecting histone fold with HEAT repeats; dashed black on blue: last two helices of HEAT repeats. **b,** TAF6L linker (in red), continuing the beta-strand complementing the the TAF5L WD40 repeat (light violet), is much shorter compared to the TAF6 linker (green). The TAF6L linker constrains the position of the HEAT repeats.

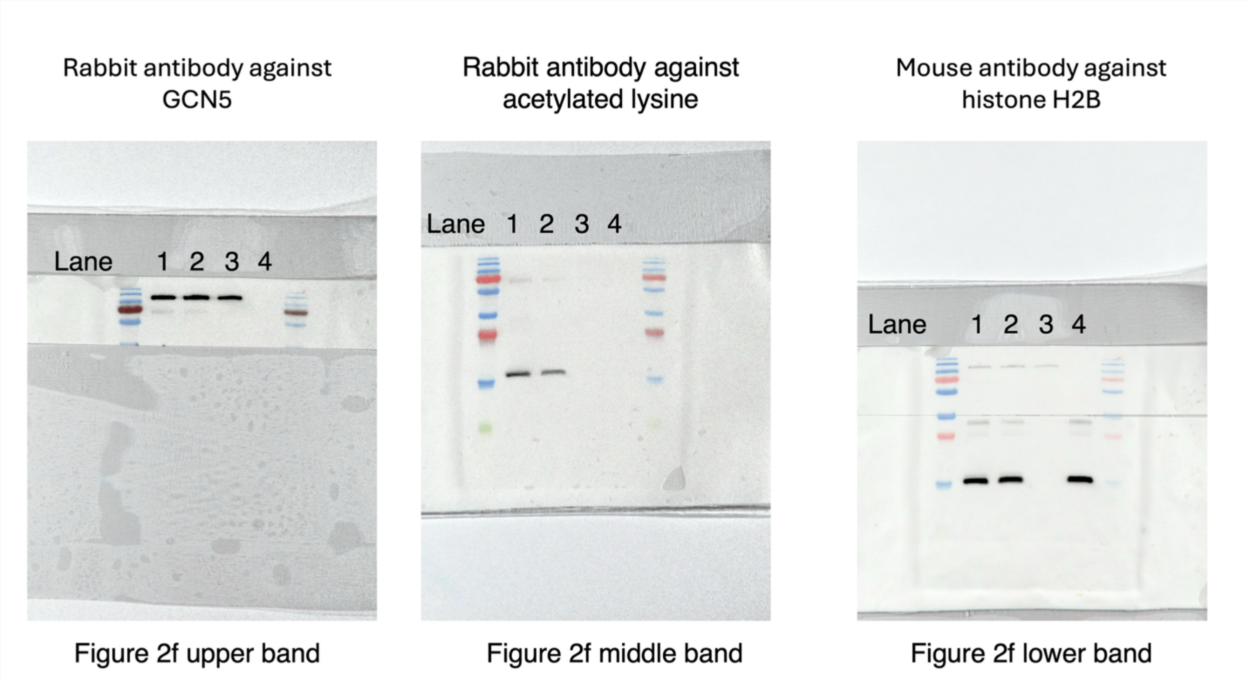

Supplementary Figure 6: Uncropped blots of Figure 2f.
The figure follows the activity of SAGA/ATAC in acetylating nucleosomes. The same membrane was analyzed by three different antibodies producing three blots as follows: Left panel. Rabbit antibody against GCN5. The membrane was physically cut to allow analysis at the same time by two different antibodies: upper part by antibody for GCN5 and lower part by antibody for histone H2B. The upper part is presented here. Middle panel. Rabbit antibody against acetylated lysine. Right panel. Mouse antibody against histone H2B in lower part of the membrane. The upper part of the membrane, analyzed with an antibody against GCN5, is also showed. Only three out of the four lanes are shown in the final figure. Lane 1 and Lane 2 show results for nucleosomes with and without a linker.

	Overall (EMDB-53935)	TRRAP (EMDB-53896)	TRRAP-end (EMDB-53932)	Core (EMDB-53895)	SF3B (EMDB-53934)	Composite (EMDB-53937) (PDB 9RDK)
**Data collection and processing**						
Magnification	165,000	165,000	165,000	165,000	165,000	165,000
Voltage (kV)	300	300	300	300	300	300
Electron exposure (e–/Å^2)	40	40	40	40	40	40
Defocus range (μm)	1.2-3.5	1.2-3.5	1.2-3.5	1.2-3.5	1.2-3.5	1.2-3.5
Pixel size (Å)	0.729	0.729	0.729	0.729	0.729	0.729
Symmetry imposed	C1	C1	C1	C1	C1	C1
Initial particle images (no.)	1,027,610	1,027,610	1,027,610	1,027,610	1,027,610	
Final particle images (no.)	272,167	272,167	272,167	59,421	59,421	
Map resolution (Å)<br>FSC threshold	2.41<br>0.143	2.38<br>0.143	3.24<br>0.143	2.58<br>0.143	2.90<br>0.143	
Map resolution range (Å)	5-2.4	5-2.4	7-3.0	5-2.4	5-2.8	7-2.4
**Refinement**						
Initial model used (PDB code)						8H7G, 7KTR,7KTS
Model resolution (Å)<br>FSC threshold						2.3<br>0.143
Map sharpening *B* factor (Å^2)	-58.8	-47.6	-100.4	-40.5	-62.4	
**Model composition**<br>Non-hydrogen atoms<br>Protein residues<br>Ligands						53,996<br>6,734<br>0
***B* factors (Å^2)**<br>Protein						88.9
**R.m.s. deviations**<br>Bond lengths (Å)<br>Bond angles (°)						0.005<br>1.064
**Validation**<br>MolProbity score<br>Clashscore<br>Poor rotamers (%)						1.35<br>1.33<br>1.54
**Ramachandran plot**<br>Favored (%)<br>Allowed (%)<br>Disallowed (%)						95.06<br>1.84<br>0.11

Supplementary Table 1. Cryo-EM data collection, refinement and validation statistics
